# LKDA-Net: Hierarchical transformer with large Kernel depthwise convolution attention for 3D medical image segmentation

**DOI:** 10.1371/journal.pone.0329806

**Published:** 2025-08-08

**Authors:** Ming Li, Jingang Ma, Jing Zhao

**Affiliations:** 1 Graduate School, Shandong University of Traditional Chinese Medicine, Jinan, China; 2 School of Medical Information Engineering, Shandong University of Traditional Chinese Medicine, Jinan, China; 3 Qilu University of Technology (Shandong Academy of Sciences), Jinan, China; Kafkas University: Kafkas Universitesi, TÜRKIYE

## Abstract

Since Transformers have demonstrated excellent performance in the segmentation of two-dimensional medical images, recent works have also introduced them into 3D medical segmentation tasks. For example, hierarchical transformers like Swin UNETR have reintroduced several prior knowledge of convolutional networks, further enhancing the model’s volumetric segmentation ability on three-dimensional medical datasets. The effectiveness of these hybrid architecture methods is largely attributed to the large number of parameters and the large receptive fields of non-local self-attention. We believe that large-kernel volumetric depthwise convolutions can obtain large receptive fields with fewer parameters. In this paper, we propose a lightweight three-dimensional convolutional network, LKDA-Net, for efficient and accurate three-dimensional volumetric segmentation. This network adopts a large-kernel depthwise convolution attention mechanism to simulate the self-attention mechanism of Transformers. Firstly, inspired by the Swin Transformer module, we investigate different-sized large-kernel convolution attention mechanisms to obtain larger global receptive fields, and replace the MLP in the Swin Transformer with the Inverted Bottleneck with Depthwise Convolutional Augmentation to reduce channel redundancy and enhance feature expression and segmentation performance. Secondly, we propose a skip connection fusion module to achieve smooth feature fusion, enabling the decoder to effectively utilize the features of the encoder. Finally, through experimental evaluations on three public datasets, namely Synapse, BTCV and ACDC, LKDA-Net outperforms existing models of various architectures in segmentation performance and has fewer parameters. Code: https://github.com/zouyunkai/LKDA-Net.

## Introduction

Since 3D medical images possess richer and more detailed spatial information than 2D images, 3D voxel segmentation is a critical technique that enables the visualization of medical images, aids in diagnosis, and facilitates the planning of treatments [1, 2]. For example, hierarchical transformer models like the Swin Transformer have been introduced into the segmentation of 3D medical images and have achieved excellent performance on multiple volumetric segmentation benchmarks [3–6]. These models have reincorporated some prior knowledge of convolutional neural networks, such as local connectivity and translation invariance, enhancing the applicability of transformers in the field of 3D medical images. Their strategy of splitting the input into patches and using the window-level self-attention mechanism to model global dependencies has further enlarged the receptive field and strengthened the feature representation [7]. Therefore, these hybrid transformer-convolution frameworks have achieved a significant improvement in performance [8].

However, directly applying 3D transformer models as general backbone networks has issues such as high computational overhead and high memory requirements [3]. The computational complexity of the global self-attention mechanism grows quadratically with the increase in input resolution [9]. High-resolution 3D medical images often contain a large amount of fine-grained information, imposing higher requirements on the feature extraction capabilities of models [10]. Therefore, the design of efficient 3D medical image analysis models still remains to be explored [11, 12]. We believe that compared with transformers, 3D convolutional networks can simulate the behavior of large receptive fields with fewer parameters through depthwise convolutions. The depthwise convolution within local regions can mimic the window-level self-attention computation of transformers [3]. Large-kernel depthwise separable convolutions can provide global-level receptive fields for feature extraction, replacing the expensive global self-attention operations of transformers [13].

Based on the above considerations, this paper proposes a lightweight three-dimensional convolutional network named LKDA-Net, which uses a large-kernel depthwise convolution attention mechanism to simulate the Transformer self-attention mechanism, aiming to achieve efficient and accurate three-dimensional volumetric segmentation. Specifically, inspired by the design of the Swin Transformer module, we have investigated large-kernel convolution attention mechanisms of different sizes to obtain a larger global receptive field. Furthermore, we use the Inverted Bottleneck with Depthwise Convolutional Augmentation to replace the MLP in the Swin Transformer module, which can enhance the feature expression ability and improve the segmentation performance while reducing the redundancy among channels. We evaluated the LKDA-Net on three public three-dimensional medical image segmentation datasets, and the results show that it outperforms current models with various architectures and has fewer parameters. Our main contributions are as follows:

- We propose the LKDA-Net, which is a lightweight convolutional network for three-dimensional medical image segmentation based on the LKDA-Net Block. The LKDA-Net Block explores large-kernel convolution attention mechanisms of different sizes to obtain a larger global receptive field. In addition, we design and use the Inverted Bottleneck with Depthwise Convolution Augmentation to replace the multi-layer perceptron (MLP) to enhance the expression of channel features, so as to reduce the number of parameters and improve the segmentation performance.
- We propose a skip connection fusion module to achieve smooth feature fusion, enabling the decoder to effectively utilize the feature information obtained by the encoder and optimize the feature processing effect.
- We evaluated the segmentation performance of our LKDA-Net on three public datasets and provided visual analysis and parameter quantity comparison. The experimental results demonstrate that our model outperforms current models with various architectures in terms of performance and has significantly fewer parameters.

## Related work

### CNN-based segmentation approaches

In the field of deep learning-based image segmentation, the primary network architectures encompass three categories: Convolutional Neural Network (CNN)-based methods, Transformer-based methods, and CNN-Transformer hybrid architectures. CNN-based methods leverage the robust spatial feature extraction capabilities of CNNs to effectively identify and segment anatomical structures in medical images. Convolutional neural networks (CNNs) [14–16] have emerged as potent means for handling medical image segmentation tasks, owing to their remarkable capacity in seizing multiscale representations, local semantic details, as well as texture particulars. Çiçek and his collaborators [17] augmented the U-Net framework by substituting 2D convolutions with 3D operations for the purpose of segmentation in volumetric images with sparse labels. Isensee *et al*.[16] put forward a nnU-Net model founded on the U-Net structure, which incorporates an automated setup to distill features from images at multiple hierarchical levels.

Furthermore, investigators have delved into the acquisition of local-global information via pure CNN architectures, such as deformable convolutions [18–20], depthwise convolutions [21–23] and large kernel convolutions [13, 24]. For example,In the detection of bipolar disorder using OCT images, Attention TurkerNeXt employs interpretable attention mechanisms and feature visualization techniques to identify critical anatomical regions that drive the model’s decision-making process[25]. Ho *et al*. [26] used relatively large 7×7 kernels within skip connections to deal with the segmentation of splenic regions. Similarly, Li *et al*. [21] proposed the Lkau-net architecture, integrating extensive depthwise convolutions and large-kernel convolutions within the decoder for medical image volume segmentation. Unfortunately, as the kernel size increases, both model parameters and FLOPs increase significantly, thereby affecting training and inference efficiency. To improve the effectiveness of large kernels, ConvNeXt [27] utilizes the potential of large kernel depthwise convolutions, a technique originating from the field of natural image processing. However, in the field of volume segmentation, the application of large-kernel depthwise convolutions is still relatively under-explored. Given the large receptive field provided by these depthwise convolutions, we believe that they have the potential to emulate Transformer behaviors, making them suitable for effective use in volume segmentation tasks.

### Transformers-based segmentation approaches

Subsequently, Transformers have achieved remarkable success in natural language processing (NLP), and Transformer-based approaches for medical image segmentation have emerged as a key research focus. By leveraging self-attention mechanisms, these methods capture global dependencies to improve the understanding and segmentation accuracy of complex medical images, particularly those with challenging backgrounds and irregular anatomical structures[28]. Recent progress in Vision Transformers [29] has overcome Long-Range dependency issues, especially in Medical Image Segmentation [6, 30]. A significant innovation in this area is the Swin-Unet [28], which has a U-shaped encoder-decoder structure enhanced with Swin Transformer blocks. Likewise, Jiang *et al*. introduced SwinBTS [31], which uses improved Transformer modules to extract detailed features. Zhou *et al*.’s nnFormer [6] retains the use of convolutional layers for local image details and a hierarchical structure for multi-scale features. However, Transformer-based volumetric segmentation models have a large number of parameters and long training times, and the high computational complexity due to multi-scale feature extraction makes the situation worse [32]. This limitation leads to a reconsideration of whether convolutional neural networks can effectively mimic Transformer advantages for efficient feature extraction.

### CNN-transformer hybrid segmentation approaches

Recently, researchers have begun exploring methods based on CNN-Transformer hybrid architectures. These hybrid approaches aim to integrate the efficiency of CNNs in processing local image features with the capability of Transformers to capture global dependencies, thereby enhancing both the efficiency and precision of medical image segmentation[33]. Some research efforts have aimed to develop hybrid architectures that combine the U-Net model with transformers [2, 5]. This combination intends to use convolutions for local feature extraction and global self-attention to capture comprehensive global-local contextual information. One such innovation is TransUNet [33], which has introduced an encoder with a hybrid CNN-Transformer architecture. This enhancement improves segmentation performance by smoothly integrating convolutional neural networks into the Transformer framework. This integration helps to effectively regain local spatial information. TransFuse [34] adopted a parallel integration approach that combines Transformers and CNNs to boost the effectiveness of seizing global information. Furthermore, UNETR [5] has introduced a new approach to semantic segmentation of medical images by using Transformers. This novel method redefines the task as a 1D sequence-to-sequence prediction problem. Another remarkable contribution is the 3D UX-NET [2], which proposes a lightweight volumetric ConvNet module with large kernel depth-wise convolutions. This module fine-tunes stratified features, ultimately leading to improved volumetric segmentation results.

### Attention mechanism

Attention mechanism enables the model to focus flexibly and specifically on the critical parts in the image. In the field of medical image segmentation, the attention mechanism encompasses two main categories: spatial attention and channel attention. Among them, the channel attention primarily concentrates on those objects of significance [35–37], while the spatial attention lays its emphasis on salient regions [38, 39]. Currently, most of the Transformer-based methods directly utilize the self-attention mechanism of Transformer to capture global feature information. For example, MedT [40] has proposed a model based on gated axial attention, which extends the current architecture by incorporating a control mechanism into the self-attention. The guided self-attention mechanism proposed by Sinha captures richer context dependencies more accurately [41]. The global spatial attention module constructed by TransAttUnet [42] successfully combines the global spatial attention with the self-attention, allowing the model to obtain long-range context interaction information. To effectively alleviate the high computational burden caused by self-attention, SegNeXt [43] proposes an efficient multi-scale convolutional attention mechanism. PraNet [44] introduces reverse attention to improve the accuracy of segmentation boundaries. However, these techniques only focus on spatially salient regions and neglect, to some extent, the attention to important objects in the channel dimension. For instance, although CBAM integrates channel and spatial information, its spatial attention is obtained through channel compression, resulting in the uniform distribution of spatial attention weights among channels [45]. MA-Unet [46] uses attention gates to properly solve the semantic ambiguity introduced by skip connections and acquires global information at different scales by means of multi-scale prediction fusion.

## Method

**Fig 1:**
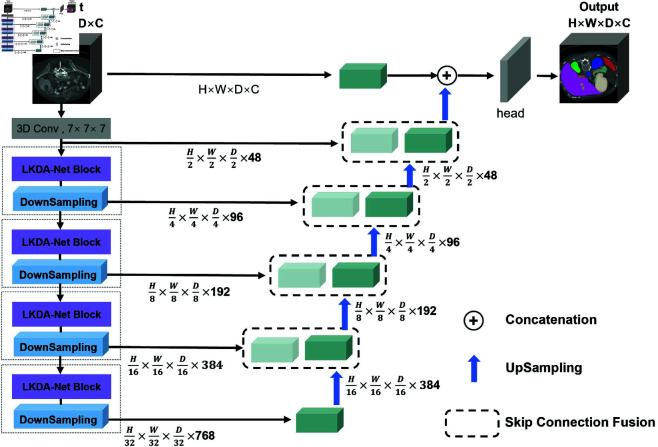
Overview of the LKDA-Net Architecture. We adopt a multi-scale hierarchical encoder-decoder structure. Firstly, the feature map is projected into multiple patches that are embedded by means of large-kernel 3D convolution. Each stage within the encoder consists of LKDA-Net Blocks and downsampling modules, which are responsible for handling feature maps at different scales. The decoder is made up of the Skip Connection Fusion Module and upsampling modules. The specific structure of the LKDA-Net Block is illustrated in Figs 2 and 3 The specific structure of the Skip Connection Fusion Module is shown in Fig 4.

Fig 1 illustrates the network architecture of our LKDA-Net constructed based on the LKDA-Net Block. Firstly, we utilize the large kernel projection to extract patch-wise features and input them into the encoder composed of the LKDA-Net Block and the downsampling block. Subsequently, the decoder consisting of the Skip Connection Fusion Module and the upsampling module further extracts features and performs upsampling. Meanwhile, the feature information at different scales of the encoder is fused through the Skip Connection Fusion Module.

For a more detailed comparison, Table 1 comprehensively illustrates the architectural differences between the proposed LKDA-Net and existing models from multiple perspectives. Transformer-based models (e.g., Swin-Unet,nnFormer ) rely on self-attention mechanisms with quadratic computational complexity $\text{O}({\text{N}}^{2})$, which hinders efficient and continuous processing of high-resolution 3D volumetric data. CNN-Transformer hybrid models (e.g., TransUNet, UNETR, Swin-UNETR) utilize Transformers for global context modeling and CNNs for local feature extraction. However, they underutilize the inherent inductive bias of convolutions and introduce redundant cross-channel parameters through MLP modules. Meanwhile, large-kernel convolutional models (e.g., MedNext) capture global information via expansive kernels but fail to explicitly model channel-wise and spatial dependencies, resulting in suboptimal segmentation performance for complex small targets. In contrast, the proposed LKDA-Net addresses these limitations through three key innovations: (1) Large Kernel Depthwise Convolution Attention (LKD Attention) enables global context modeling with linear complexity *O*(*N*), preserving 3D spatial continuity while expanding the effective receptive field; (2) Inverted Bottleneck with Depthwise Convolution Enhancement (DWCA) refines local feature representation by decoupling channel interactions and eliminating redundancy; (3) A Group Convolution-based Skip Connection Fusion Module aligns multi-scale encoder-decoder features, effectively mitigating semantic gap issues caused by resolution mismatches.

**Table 1:** Architectural Comparison of LKDA-Net with State-of-the-Art Models.

Model	Global Modeling Strategy	Local Feature Extraction	Feature Fusion Strategy	Parameter Efficiency Optimization	Core Differences vs LKDA-Net
Swin-Unet	Windowed Self-Attention (W-MSA)	Multilayer Perceptron (MLP)	Standard Skip Connections	Window Partitioning for Reduced Computation	High computational complexity, relies on self-attention
nnFormer	Hierarchical Self-Attention (LV-MSA)	MLP	Multi-scale Feature Concatenation	Optimized Attention for Efficiency	Large parameter count, long training time
TransUNet	Transformer Decoder	CNN Encoder	Hybrid CNN-Transformer Feature Fusion	Feature Map Downsampling	Underutilized convolutional inductive bias
UNETR	Pure Transformer Encoder	3D Convolutional Decoder	Cross-scale Feature Concatenation	Reduced Input Resolution for Efficiency	High memory consumption, poor scalability for high-resolution data
Swin-UNETR	3D Swin Transformer (Shifted Window)	Depthwise Conv + MLP	Standard Skip Connections	Hierarchical Downsampling + Window Attention	Still relies on high-complexity Transformer operations
MedNext	Large-kernel Convolution for Receptive Field	MLP	Multi-path Feature Fusion	Dynamic Kernel Size Adjustment	No explicit channel/spatial dependency modeling
**LKDA-Net (Ours)**	**Large Kernel Depthwise Conv Attention (LKD Attention)**	**Inverted Bottleneck with Depthwise Conv Augmentation (DWCA)**	**Skip Fusion Module (Grouped Convolution)**	**Depthwise Separable Conv + Channel-wise Compression**	**Balanced lightweight design, effective global-local fusion**

### LKDA-Net block

**Fig 2:**
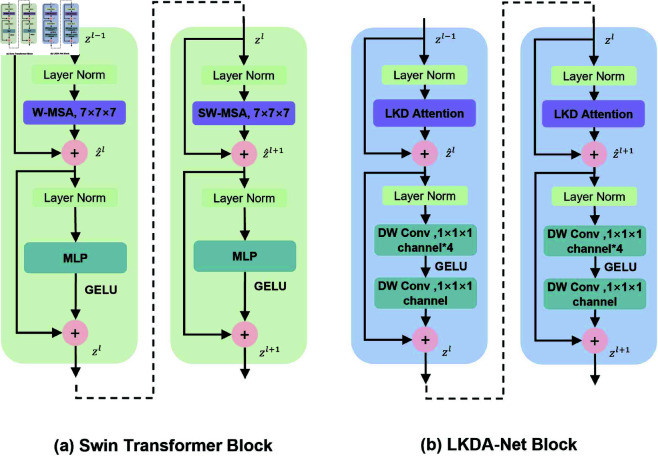
(a) Architecture of the Swin Transformer Block. (b) Architecture of the LKDA-Net Block proposed by us. Compared with the Swin Transformer block, the LKDA-Net Block obtains a larger receptive field through Large Kernel Depthwise Convolution Attention (LKD Attention) and Inverted Bottleneck with Depthwise Convolutional Augmentation (DWCA), while also improving the quality of feature representation.

Inspired by ConvNeXt and Swin Transformers, we propose the LKDA-Net Block for 3D medical image segmentation. As directly applying transformers as a universal backbone has the problem of high computational complexity, we propose to simulate the self-attention mechanism of transformers for global relationship modeling based on the large kernel depthwise convolution attention mechanism, so as to efficiently extract global features. Furthermore, we have designed an efficient 3D medical image segmentation network. Fig 2 compares the differences between the Swin Transformer Block and our proposed LKDA-Net Block. The distinct designs of the LKDA-Net Block mainly include Large Kernel Depthwise Convolution Attention (LKD Attention) and Inverted Bottleneck with Depthwise Convolution Augmentation (DWCA).

#### Large Kernel Depthwise Convolution Attention (LKD Attention).

Swin Transformer employs Window-based Multi-head Self-Attention (W-MSA) to capture local dependencies and further utilizes Shifted Window MSA to explore the dependencies among different windows, thereby obtaining a global receptive field. We have found that there is a significant similarity between the per-channel computation of Depthwise Convolution and the weighted summation of self-attention. We believe that using Large Kernel Depthwise Convolution Attention (LKD Attention) can acquire a large receptive field just like MSA does, thus capturing global dependencies.

The LKD Attention proposed by us adaptively utilizes channel-level and spatial-level contextual information through a large receptive field. Specifically, multiple large kernel depthwise convolutions are employed to extract multi-scale features. Moreover, we cascade these large kernel depthwise convolutions, endowing them with increasing dilation rates and growing kernel sizes. On the one hand, this design can recursively aggregate contextual information within the receptive field. On the other hand, the features extracted within deeper and larger receptive fields contribute more to the output, enabling the LKD Attention to capture more effective features.

**Fig 3:**
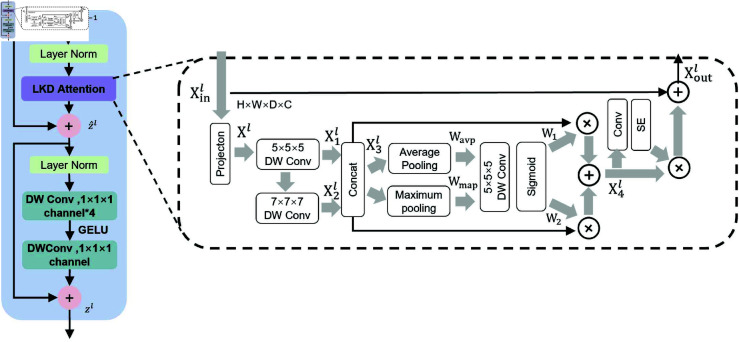
Details of the LKD Attention within the proposed LKDA-Net Block.

The LKD Attention is shown in Fig 3. In the specific operations of the LKD Attention, we first utilize a 1×1×1 convolutional layer to conduct a projection operation that halves the number of channels, aiming to reduce the complexity caused by multiple convolutions. The input feature $X_{in}^{l}\in R^{H\;\times \;W\;\times \;D\;\times \;C}$ of *l* layer is projected to the layer $X^{l}\in R^{H\;\times \;W\;\times \;D\;\times \;\frac{C}{2}}$ (where *C* represents the number of channels, and *H*, *W*, *D* are the dimensions of the 3D image). Secondly, we perform depthwise convolution operations (DW Conv) with two large kernels respectively on the projected feature maps. The first one is a depthwise convolution with a dilation rate of 1 and a kernel size of 5×5×5, and the second one is a depthwise convolution with a dilation rate of 3 and a kernel size of 7×7×7. The large convolutional kernels can capture the global feature dependencies within a local region, simulating the window-level self-attention calculation in transformers. Meanwhile, the channel independence of depthwise convolutions is also similar to the operations of self-attention on each patch.

$$X_{l}=\text{Project}(X_{in}^{l})$$ (1)

$$X_{1}^{l}=\text{DW Conv}(5,1)(X_{l})$$ (2)

$$X_{2}^{l}=\text{DW Conv}(7,3)(X_{1}^{l})$$ (3)

By cascading the depthwise convolutions with two large kernels, the LKD Attention can obtain an effective receptive field with a size of 23×23×23. The two resulting feature maps $X_{1}^{l}\in R^{H\;\times \;W\;\times \;D\;\times \;\frac{C}{2}}$ and $X_{2}^{l}\in R^{H\;\times \;W\;\times \;D\;\times \;\frac{C}{2}}$ are concatenated to restore the original number of channels, obtaining the feature $X_{3}^{l}\in R^{H\;\times \;W\;\times \;D\;\times \;C}$. Then, the global spatial relationships of these features are effectively modeled by applying average pooling and maximum pooling along the channels of the feature $X_{3}^{l}$.

$$X_{3}^{l}=\text{Concat}([X_{l}^{1};X_{l}^{2}])$$ (4)

$$w_{avp}=\text{Average Pooling}(X_{3}^{l})$$ (5)

$$w_{map}=\text{Maximum pooling}(X_{3}^{l})$$ (6)

Then, a depthwise convolution with a size of 5×5×5 is used to mix and interact the information obtained in the previous step among different spatial feature representations. Finally, the Sigmoid activation function is employed to obtain the weight values *w*_1_ and *w*_2_.

$$\left[w_{1};\;w_{2}]=\text{Sigmoid}(\text{Conv}5([w_{avp};\;w_{map}])\right)$$ (7)

These weight values are utilized to adaptively select the features from different large kernels and calibrate them to obtain $X_{4}^{l}$.

$$X_{4}^{l}=(w_{1}⊗X_{1}^{l})⊕(w_{2}⊗X_{2}^{l})$$ (8)

Subsequently, after performing a 1×1×1 convolution operation on the obtained $X_{4}^{l}$, a 5×5×5 SE module is used to weight the feature map, thereby explicitly modeling the interdependencies among the channels of its convolutional features to improve the quality of the feature map representation. Finally, the residual connection is utilized to generate the output feature $X_{out}^{l}\in R^{H\;\times \;W\;\times \;D\;\times \;C}$.

$$w_{3}=\text{Conv}1(\text{SE}(X_{4}^{l}))$$ (9)

$$X_{out}^{l}=w_{3}⊗X_{4}^{l}+X_{in}^{l}$$ (10)

#### Inverted Bottleneck with Depthwise Convolution Augmentation (DWCA).

In the Transformer structure, an inverted bottleneck is designed, that is, the dimension of the hidden layer in the MLP module is four times wider than the input dimension. MobileNetV2 [47] first applied the inverted bottleneck structure to Convnet, and then several advanced Convnet models also adopted a similar design. Therefore, we adopt a similar inverted bottleneck structure and use the Depthwise Convolution with a size of 1 × 1 × 1 to process the features. Inspired by vision transformers, instead of using the batch normalization (BN) commonly used in Convnet, we use Layer Normalization (LN) for the normalization operation. In addition, we replace the RELU activation function with the smoother GELU activation function.

In the Inverted Bottleneck structure we proposed, the features passing through the Layer Normalization layer are first expanded to four times the number of input channels through Depthwise Convolution. After the result is processed by the GELU activation function, the Depthwise Convolution with a size of 1 × 1 × 1 is then used to independently scale each channel feature back to the original number of input channels. Subsequently, a residual connection is made with the original feature map to obtain the output of the Inverted Bottleneck. We expand and compress the dimensions of each channel in an independent manner, which reduces the redundancy among channels while enhancing the feature expression ability. Therefore, we define the outputs of *l* and l+1 layer of the LKDA-Net Block as follows:

$${\hat{z}}^{l}=z^{l-1}+\text{DW Conv}(\text{LN}(z^{l-1}))+\text{SE}(\text{LN}(z^{l-1}))$$ (11)

$$z^{l}={\hat{z}}^{l}+\text{DWCA}(\text{LN}({\hat{z}}^{l}))$$ (12)

$${\hat{z}}^{l+1}=z^{l}+\text{DW Conv}(\text{LN}(z^{l}))+\text{SE}(\text{LN}(z^{l-1}))$$ (13)

$$z^{l+1}={\hat{z}}^{l+1}+\text{DWCA}(\text{LN}({\hat{z}}^{l+1}))$$ (14)

where prediction ${\text{z}}^{\text{l}}$ and prediction *z*^*l* + 1^ are the outputs of different depth layers of DWConv. SE represents Squeeze-and-Excitation block. LN denotes layer normalization, and DWCA represents Inverted Bottleneck with Depthwise Convolution Augmentation.

### LKDA-Net encoder

Take a 3D voxel data from the training set as the input of the encoder. Our encoder is divided into five stages. In the first stage, instead of adopting the method of linear projection embedding, we use a Depthwise Convolution layer with a size of 7×7×7 to perform patch embedding, obtaining a feature map with a resolution of $\frac{H}{2}\;\times \;\frac{W}{2}\;\times \;\frac{D}{2}\;\times \;48$. This patch-based feature extraction aims to simulate the operation of first performing patch segmentation and then learning embeddings in visual transformers. Compared with the global self-attention in transformers which has high computational cost, our design provides an efficient alternative to capture global dependencies within local regions. The remaining four stages are each composed of the LKDA-Net Block and downsampling. In the LKDA-Net Block, the Large Kernel Depthwise Convolution Attention is used to model global dependencies, followed by the Inverted Bottleneck with Depthwise Convolutional Augmentation structure which expands and compresses the dimensions of each channel in an independent manner, enhancing the feature expression ability while reducing the redundancy among channels. Instead of using MLP, we use a 3D convolution with a stride of 2 and a convolution kernel size of 2×2×2 to halve the resolution of the feature map so that the information of each channel can be better fused. The third, fourth, and fifth stages follow the same operations, resulting in feature map resolutions of $\frac{H}{8}\;\times \;\frac{W}{8}\;\times \;\frac{D}{8}\;\times \;192$, $\frac{H}{16}\;\times \;\frac{W}{16}\;\times \;\frac{D}{16}\;\times \;384$, and $\frac{H}{32}\;\times \;\frac{W}{32}\;\times \;\frac{D}{32}\;\times \;768$, respectively. Multiple-scale hierarchical feature representations are extracted at each stage and are further utilized in the segmentation of 3D voxel data.

### LKDA-Net decoder

Our decoder utilizes the Skip Connection Fusion Module to further extract hierarchical visual feature representations and optimize the segmentation results. Specifically, firstly, the feature outputs of each encoder stage are concatenated and fused with the upsampled decoding features. Secondly, the results of multi-step fusion are input into a convolutional layer with a softmax activation function to predict the final segmentation probabilities. The application of the Skip Connection Fusion Module in the decoder enables our model to align the semantic information of features at different resolutions, so as to optimize the segmentation of fine-grained volumetric data.

#### Skip connection fusion module.

**Fig 4:**
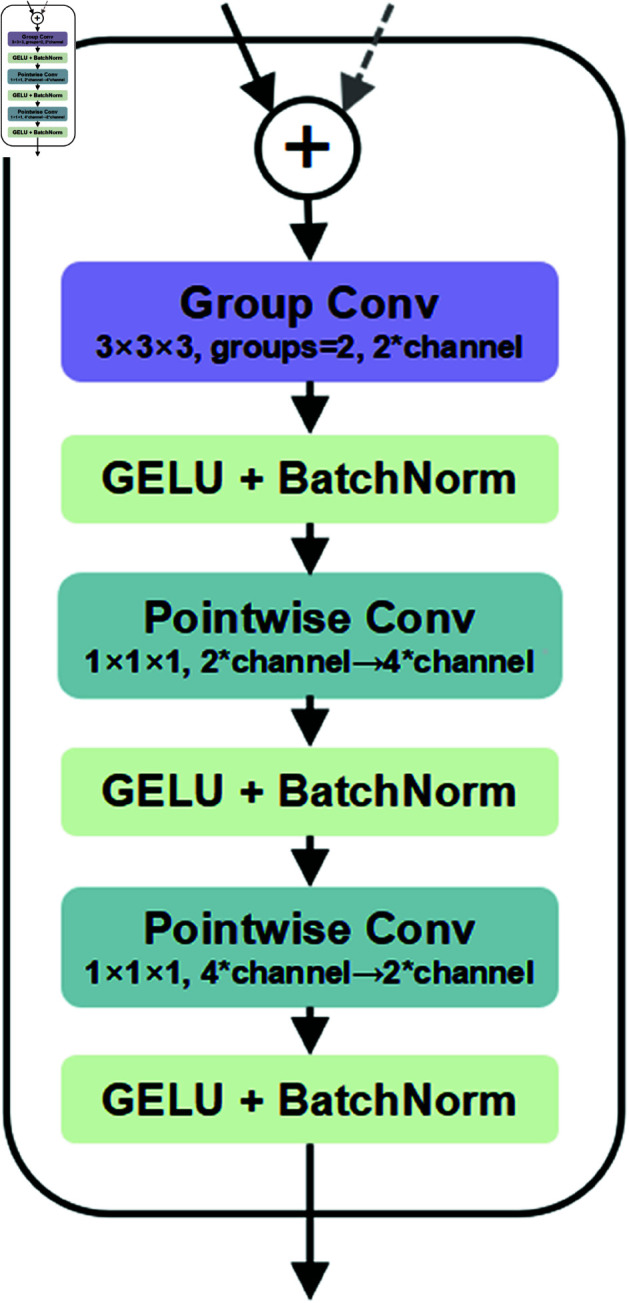
The structure of the Skip Connection Fusion Module. The module employs group convolution to separately process encoder and decoder features, followed by inverted bottleneck pointwise convolutions for adaptive fusion.

Traditional skip connections usually utilize ordinary convolution operations for feature fusion. Such operations can be straightforward and lack flexibility and pertinence during the processing, causing the encoder and decoder to bear more computational loads and data processing pressures. However, the skip connection fusion module we propose adopts group convolution as a key component to effectively address these problems. As shown in Fig 4, we divide the convolution operation into two groups. One group is specifically dedicated to the refined extraction of “feature-to-feature” for the features from the encoder in the skip connection, while the other group is responsible for carrying out the same operation on the decoder features after upsampling. Here, the kernel size of the group convolution is set to 3 × 3 × 3, the stride is 1, and the padding is 1. In order to achieve a more sufficient and comprehensive feature fusion effect, we add two inverted bottleneck pointwise convolutions after the group convolution operation is completed. The skip connection fusion module can reasonably and adaptively allocate the features before fusion to the group convolution for processing according to the characteristics of the features themselves. Subsequently, the highly efficient and dense pointwise convolutions play a crucial role in the entire feature fusion process and undertake the main task of feature fusion.

Moreover, inside the skip connection fusion module, each convolution operation is followed by a GELU activation function layer and a BatchNorm normalization layer, so as to further optimize the feature processing effect and enhance the stability and adaptability of the module. The definition of the Skip Connection Fusion Module Block is as follows:

$$f_{c}=Concat(BN(Conv(f_{E})),BNConv((f_{D})))$$ (15)

$$f_{fusion}^{\prime }=BN(\sigma_{1}(PointwiseConv(f_{c})))$$ (16)

$$f_{fusion}=BN(\sigma_{1}(PointwiseConv(f_{fusion}^{\prime })))$$ (17)

where *f*_*E*_ and *f*_*D*_ are the features of the encoder and the decoder respectively. *f*_*c*_ is the concatenated feature, and *f*_*fusion*_ is the output fused feature map of the Skip Connection Fusion Module.

#### Upsampling block.

The Upsampling Block is mainly constituted by an upsampling layer, a convolution layer, a batch normalization layer, and a ReLU activation function. Specifically, for the upsampling process, we employ the bilinear interpolation method to upscale the feature map by a factor of 2. This technique is beneficial as it can enhance the resolution of the feature map in a relatively smooth and efficient manner. The convolution layer within the block has a kernel size of 3×3×3, a stride of 1, and a padding of 1. Such convolution settings enable the extraction and refinement of local features. Through the combined utilization of these techniques, namely the bilinear interpolation, the convolution operation, the batch normalization, and the ReLU activation, we are capable of not only effectively increasing the resolution of the feature map but also retaining the essential features.

### Loss function

To calculate the loss between the predicted 3D voxels and the ground truth, we utilize a combination of cross-entropy loss and soft dice loss, leveraging the advantages of both loss functions. The loss function for MedX-Net is defined as follows:

$$ℒ(Z,P)=1-\sum_{j=1}^{N}(\frac{2*\sum_{i=1}^{I}Z_{i,j}\cdot P_{i,j}}{\sum_{i=1}^{I}Z_{i,j}^{2}+\sum_{i=1}^{I}P_{i,j}^{2}}+\sum_{i=1}^{I}Z_{i,j}\log P_{i,j})$$ (18)

where *i* represents the total number of 3D voxels, and *N* is the number of predicted classes. *Z*_*i*,*j*_ denotes the ground truth value of class j at voxel i, while *P*_*i*,*j*_ represents the predicted probability output of class j at voxel i by the model.

## Experiments and results

### Datasets

Our method was evaluated on three datasets: Synapse, BTCV, and ACDC, with both quantitative metrics and visual analysis results presented. These datasets cover diverse anatomical regions including abdominal multi-organ and cardiac regions, and incorporate imaging modalities such as CT and MRI, enabling comprehensive validation of the model’s segmentation performance and generalization capabilities. As widely recognized and extensively utilized benchmarks in the medical image segmentation community, these datasets provide a reliable reference standard to ensure the credibility and comparability of our experimental findings.

**Synapse Dataset.** The Synapse dataset holds a significant position in the field of medical image analysis. It mainly focuses on CT scan data of abdominal organs. The data is sourced from a specific group of 30 patients. The data partitioning approach is inspired by the concept of TransUnet. Specifically, 18 groups of data are allocated to the training set to facilitate the model’s learning and training procedures, which allows the model to acquire the normal and abnormal feature patterns of abdominal organs. The remaining 12 groups of data function as the test set, aiming to assess the performance and accuracy of the trained model and examine the model’s generalization capability on unseen data. This dataset encompasses the delineation of eight distinct organs, specifically the spleen, left kidney, pancreas, stomach, aorta, liver, gallbladder, and right kidney.

**BTCV Dataset.** The BTCV Dataset (Beyond-the-Cranial-Vault Abdominal CT Organ Segmentation) comprises 30 training instances and 20 testing instances. Notably, this dataset not only encompasses the eight organs present in the Synapse dataset but also encompasses structures of the esophagus, inferior vena cava, portal and splenic veins, as well as the right and left adrenal glands.

**ACDC Dataset.** The ACDC Dataset (Automated Cardiac Diagnosis Challenge) comprises MRI scan images from numerous patients, featuring a composition of 70 training samples, 10 validation samples, and 20 test samples. Within each MRI image, delineations are made for the myocardium (MYO), right ventricle (RV), and left ventricle (LV) regions.

### Implementation details

Our developmental environment consists of Ubuntu 18 and PyTorch 1.12. We engage in training utilizing a singular GeForce RTX 3090 endowed with 24GB. The orchestration employs the AdamW optimizer, coupled with a stipulation of a maximum iteration count amounting to 40000, and an initial learning rate instantiation set at 0.0001. As a preliminary step, all images undergo resampling to conform to a uniform voxel spacing and are subsequently cropped to dimensions of 96×96×96. As the training regimen unfolds, a symphony of data augmentation methodologies is invoked. Within this repertoire, the repertoire includes scaling, rotation, luminance and contrast modulations, Gaussian noise infusions, as well as Gaussian blurring. For the purpose of experimental evaluation, the Average DSC is harnessed as the yardstick of assessment. Additionally, we employ the Hausdorff Distance 95% (HD95) metric to measure the maximum boundary error of segmentation results, which further reveals the model’s sensitivity to anatomical boundaries. A lower HD95 value indicates higher agreement between the segmentation boundaries and the ground truth annotations. The ground truth and predicted values are denoted by $z_{i}\in Z$ and $p_{i}\in P$, respectively, for a given semantic class *i*. The ground truth and predicted surface point sets are denoted by $z^{\prime }\in Z^{\prime }$ and $p^{\prime }\in P^{\prime }$, respectively. The DSC metric is defined as:

$$DSC(Z,P)=\frac{2\sum_{i=1}^{I}z_{i}p_{i}}{\sum_{i=1}^{I}z_{i}+\sum_{i=1}^{I}p_{i}}$$ (19)

During the course of the training endeavor, we incorporated a mechanism of profound supervision. To be specific, we employed the cross-entropy loss function and the Dice loss function as the ultimate measures of loss. The computation of loss was executed by contrasting the upsampled images from distinct resolutions of the decoder with their corresponding ground truth values. Consequently, the ultimate objective function for training materialized as the summation of losses across five distinct resolutions:

$$L_{\text{all}}\;=\;\alpha_{1}L_{\left\{H,W,D\right\}}+\alpha_{2}L_{\left\{\frac{H}{2},\frac{W}{2},\frac{D}{2}\right\}}+\alpha_{3}L_{\left\{\frac{H}{4},\frac{W}{4},\frac{D}{4}\right\}}\;+\alpha_{4}L_{\left\{\frac{H}{8},\frac{W}{8},\frac{D}{8}\right\}}+\alpha_{5}L_{\left\{\frac{H}{16},\frac{W}{16},\frac{D}{16}\right\}}$$ (20)

where $\left(\alpha_{1},\alpha_{2},\alpha_{3},\alpha_{4},\alpha_{5}\right)$ respectively denote the magnitudes of loss weights for distinct resolution features. In this context, a greater weight is assigned to the loss of feature maps with higher resolutions, thus facilitating accelerated convergence and superior segmentation outcomes. We established that $\alpha_{2}=\frac{\alpha_{1}}{2},\alpha_{3}=\frac{\alpha_{2}}{2},\alpha_{4}\;=\;\frac{\alpha_{3}}{2}\;and\;\alpha_{5}=\frac{\alpha_{4}}{2}$, with the cumulative summation of all loss weight factors equating to 1.

### Comparison with state-of-the-arts

We evaluated the performance of LKDA-Net on three segmentation datasets. These segmentation datasets vary in complexity, the number of structures to be segmented, image modalities (CT, MRI), and spatial and phenotypic heterogeneity. This experimental design emphasizes the experimental effect and generalization ability of LKDA-Net in different segmentation tasks. For a detailed evaluation and comparison, we compared the performance with various recent state-of-the-art (SOTA) segmentation models. These models were trained according to the optimal parameters provided in their papers and evaluated through the same 5-fold cross-validation.

- CNN-based methods: U-Net [48], nnUNet [16].
- Transformer-based methods: Swin-Unet [28], MISSFormer [30], nnFormer [6].
- Hybrid CNN-Transformer-based methods: TransUNet [33], UNETR [5], swin-UNETR [9].
- Large convolutional kernel-based methods: 3D UX-NET [2], MedNext [11].

Table 2, Table 3 and Table 4 respectively present the performance comparisons on the Synapse multi-organ segmentation dataset, the Automated Cardiac Diagnosis dataset and the BTCV abdominal multi-organ segmentation task. When evaluating the model’s efficiency metrics, we conducted experiments under the same experimental environment and preprocessing pipeline as during the training phase. Table 5 presents the comparative results of parameter counts and computational costs across different models, where the inference time corresponds to the total time required by the model to segment 12 test samples from the Synapse dataset.

**Table 2:** The performance comparison between LKDA-Net and other existing methods on the Synapse dataset. The bold data are the optimal ones in single-organ segmentation. Note: Spl: spleen, RKid: right kidney, LKid: left kidney, Gal: gallbladder, Eso: esophagus, Liv: liver, Sto: stomach, Aor: aorta IVC: inferior vena cava, PSV: portal and splenic veins, Pan: pancreas.

Methods	Synapse								Average	
	Spl	RKid	LKid	Gal	Liv	Sto	Aor	Pan	DSC(%)	HD95 (mm)
U-Net	86.67	68.60	77.77	69.72	93.43	75.58	89.07	53.98	76.85	-
TransUNet	85.08	77.02	81.87	63.16	94.08	75.62	87.23	55.86	77.49	31.69
Swin-Unet	90.66	79.61	83.28	66.53	94.29	76.60	85.47	56.58	79.13	21.55
UNETR	85.00	84.52	85.60	56.30	94.57	70.46	89.80	60.47	78.35	18.59
MISSFormer	91.92	82.00	85.21	68.65	94.41	80.81	86.99	65.67	81.96	18.20
Swin-UNETR	95.37	86.26	86.99	66.54	95.72	77.01	91.12	68.80	83.48	10.55
3D UX-NET	95.01	85.76	87.59	67.34	94.82	80.01	90.72	80.71	85.26	11.23
nnFormer	90.51	86.25	86.57	70.17	**96.84**	**86.83**	92.04	**83.35**	86.57	10.63
LKDA-Net(Ours)	**95.63**	**87.08**	**87.39**	**71.08**	96.64	85.30	**92.67**	81.86	**87.21**	**7.33**

**Table 3:** Comparative performance analysis of our LKDA-Net with other existing methods on ACDC dataset. Best results are in bold. Note: RV: Right ventricle, Myo: Myocardium, LV: Left ventricle.

Methods	ACDC			
	RV	Myo	LV	Average DSC(%)
U-Net	87.10	80.63	94.92	87.55
TransUNet	88.86	84.54	95.73	89.71
Swin-Unet	88.55	85.62	95.83	90.00
UNETR	85.29	86.52	94.02	88.61
MISSFormer	86.36	85.75	91.59	87.90
nnFormer	90.94	89.58	95.65	92.06
LKDA-Net(Ours)	**91.75**	**90.60**	**96.02**	**92.79**

**Table 4:** Comparison results on the BTCV dataset. Note: Spl: spleen, RKid: right kidney, LKid: left kidney, Gal: gallbladder, Eso: esophagus, Liv: liver, Sto: stomach, Aor: aorta IVC: inferior vena cava, PSV: portal and splenic veins, Pan: pancreas, RAG: adrenal gland, LAG: Left adrenal gland.

Methods	Spl	RKid	LKid	Gal	Eso	Liv	Sto	Aor	IVC	PSV	Pan	RAG	LAG	DSC	HD95
U-Net	90.68	82.62	85.05	57.33	70.11	93.44	73.14	84.54	77.33	70.17	65.06	65.95	62.25	75.21	-
nnUNet	95.95	88.35	93.02	70.13	76.72	96.51	**86.79**	88.93	82.89	**78.51**	**79.60**	73.26	68.35	83.16	-
TransUNet	94.55	89.20	90.97	68.38	75.61	96.44	83.52	88.55	82.48	74.21	76.02	67.23	67.03	81.31	12.98
UNETR	90.48	82.51	86.05	58.23	71.21	94.64	72.06	86.57	76.51	70.37	66.06	66.25	63.04	76.00	8.82
Swin-UNETR	94.59	88.97	92.39	65.37	75.43	95.61	75.57	88.28	81.61	76.30	74.52	68.23	66.02	80.44	13.49
MedNext	95.59	88.76	92.87	66.42	75.52	95.81	76.87	88.52	82.01	76.21	76.82	70.31	66.53	80.94	9.34
nnFormer	94.58	88.62	93.68	65.29	76.22	96.17	83.59	**89.09**	80.80	75.97	77.87	70.20	66.05	81.62	5.15
LKDA-Net(Ours)	**95.97**	**91.21**	**93.71**	**70.54**	**77.07**	**96.84**	86.14	89.02	**83.07**	77.92	78.41	**73.45**	**68.64**	**83.23**	**4.85**

**Table 5:** The comparison of the LKDA-Net model with other models in terms of the number of model parameters, computational complexity (FLOPs) and Inference Time (s).

Methods	Params(M)	FLOPs(G)	Inference Time (s)
TransUNet	96.07	88.91	2.15
UNETR	92.49	75.76	1.98
Swin-UNETR	62.83	384.2	3.72
nnFormer	150.5	213.4	4.05
nnUNet	68.38	357.13	3.41
3D UX-NET	53.01	632.33	3.88
MedNext	11.65	178.05	1.28
LKDA-Net(Ours)	**48.52**	**418.52**	1.65

**Synapse Dataset:** LKDA-Net achieved an overall average Dice Similarity Coefficient (DSC) of 87.21% on the Synapse Dataset, demonstrating the best and most robust overall performance compared with other state-of-the-art (SOTA) methods. Specifically, compared with the baseline model U-Net based on Convolutional Neural Network (CNN), which achieved an average DSC of 76.85%, LKDA-Net achieved a superior overall segmentation performance. Compared to MedNext, which employs large-kernel pure convolutions to expand the receptive field, its lack of explicit modeling of channel-wise and spatial dependencies limits segmentation accuracy for small targets (e.g., adrenal glands). In contrast, LKDA-Net leverages an Inverted Bottleneck with Depthwise Convolution Enhancement (DWCA ) to enable the model to concentrate on critical anatomical regions. In addition, the average DSC obtained by LKDA-Net was also significantly higher than that of methods based purely on Vision Transformer, such as Swin-Unet, MISSFormer, and nnFormer. Although nnFormer had a lower computational complexity, LKDA-Net achieved better segmentation results while reducing the number of model parameters by two-thirds.

When LKDA-Net was compared with hybrid CNN-Transformer methods that had already reached the state-of-the-art level in various segmentation tasks, it achieved a superior overall performance. Although the UNETR had lower floating-point operations, LKDA-Net achieved a higher average DSC in terms of overall segmentation performance. While Swin-UNETR captures long-range dependencies through windowed self-attention (W-MSA), its computational complexity grows quadratically with input resolution, and its MLP modules introduce redundant cross-channel parameters. In contrast, LKDA-Net’s LKD Attention significantly mitigates boundary ambiguity (Fig 6) and demonstrates superior segmentation performance without relying on the high computational costs associated with Transformer architectures. 3D UX-NET proposed using large-kernel convolutional modules to replace Transformer modules. However, LKDA-Net achieved superior performance with a much lower computational complexity than it, which proved the effectiveness and efficiency of the LKDA-Net Block and the overall architecture we proposed. While MedNext’s large-kernel pure convolutional design yields lower computational complexity and shorter inference time compared to LKDA-Net, LKDA-Net achieves significantly higher segmentation accuracy. LKDA-Net achieves the lowest HD95 of 7.33 mm, significantly outperforming Swin-UNETR (10.55 mm) and 3D UX-NET (11.23 mm). This indicates our model not only improves volumetric overlap but also reduces extreme segmentation errors at organ boundaries. The average DSC of LKDA-Net in all organ-specific segmentation tasks had been significantly improved, indicating that LKDA-Net demonstrated excellent capabilities in feature extraction and representation from different organs.

It can be seen from Table 5 that the number of parameters of the LKDA-Net model is only 44.52 million. Compared with other pure Transformer models, the number of parameters of our model has been significantly reduced. The implementation of LKD Attention within the LKDA-Net Block substantially reduces the model’s parameter count. However, the computationally intensive nature of LKD Attention results in relatively higher FLOPs for our model. This design ensures efficient segmentation while mitigating the risks of overfitting from excessive model parameters and the overconsumption of computational resources. In terms of inference time, LKDA-Net achieves an end-to-end inference time (encompassing data preprocessing, GPU computation, and data transfer) of 1.65 seconds for segmenting 12 test samples, which is notably faster than Swin-UNETR (3.72 seconds) and nnUNet (3.41 seconds), despite their similar FLOPs. Furthermore, compared to 3D UX-NET and MedNext, which rely on large-kernel pure convolutional operations, these models exhibit comparable parameter counts to LKDA-Net but fall short in overall accuracy and inference efficiency.

**ACDC Dataset**: LKDA-Net achieved an overall average DSC of 92.79% on the ACDC dataset, demonstrating outstanding and extremely robust overall performance compared with other state-of-the-art (SOTA) methods. Specifically, compared with the U-Net baseline model based on the Convolutional Neural Network, which achieved an average DSC of 87.55%, LKDA-Net achieved a more excellent overall segmentation performance. In addition, the average DSC of LKDA-Net was significantly higher than that of methods based on pure Vision Transformer, such as Swin-Unet and MISSFormer. Even though nnFormer is currently the best-performing method on the ACDC dataset, LKDA-Net still surpassed it in terms of the overall number of parameters and overall segmentation performance. When LKDA-Net was compared with various hybrid CNN-Transformer methods, it also achieved a superior overall performance. Although models such as TransUNet, Swin-Unet, and UNETR have their own advantages in certain aspects. For example, TransUNet has a relatively low number of parameters and computational complexity, Swin-Unet performs fairly well in some indicators, and UNETR has a lower number of floating-point operations, LKDA-Net achieved a higher average DSC in terms of overall segmentation performance. Moreover, compared with the current mainstream models, the average DSC of LKDA-Net in the segmentation tasks of specific regions such as the right ventricle (RV), myocardium (Myo), and left ventricle (LV) has been significantly improved. This indicates that LKDA-Net demonstrates extremely excellent capabilities in feature extraction and representation from different cardiac structures, and it can divide different regions in cardiac magnetic resonance images more accurately, thereby providing more reliable and precise data support for cardiac-related medical research or clinical diagnosis.

**BTCV Dataset**: LKDA-Net also demonstrates excellent segmentation performance on the BTCV dataset. The current excellent pure Convolutional Neural Network model, nnUNet, has an average DSC of 83.16%, which is the best-performing model among all current mainstream models. LKDA-Net has an average DSC of 83.23% on the BTCV dataset and outperforms the nnUNet model with a lower number of model parameters and a higher average DSC. Compared with the method based on pure Vision Transformer, nnFormer, LKDA-Net has a significant advantage in average DSC and can capture the feature information in images more effectively, thus transforming it into more accurate segmentation results. When compared with various hybrid CNN-Transformer methods, LKDA-Net also stands out. While TransUNet integrates CNNs and Transformers, its encoder-decoder framework employs a simplistic fusion strategy (direct concatenation of features), leading to insufficient alignment between shallow local features and deep semantic information. In contrast, LKDA-Net achieves more precise segmentation through a skip-connection fusion module (combining grouped convolutions with inverted bottleneck pointwise convolutions). Although TransUNet and UNETR has relatively lower floating-point operations, LKDA-Net achieves a higher value in overall segmentation performance. Notably, LKDA-Net attains an HD95 of 4.85 mm, surpassing nnFormer (5.15 mm) and MedNext (9.34 mm). The reduced HD95 demonstrates the effectiveness of our skip connection fusion module in preserving anatomical details. When handling various tissue and organ segmentation tasks in the BTCV dataset, LKDA-Net can outline the target regions more accurately, and its overall segmentation effect surpasses that of the current mainstream baseline models.

**Fig 5:**
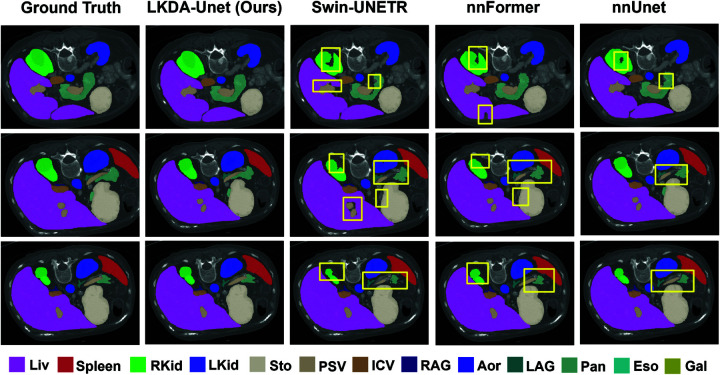
The visual segmentation effect of LKDA-Net on the Synapse dataset. The parts where our model outperforms other models are marked in yellow. Compared to other models, LKDA-Net demonstrates higher structural continuity in segmenting complex organ boundaries, such as the pancreas and kidneys, achieving results closer to the ground truth.

Fig 5 presents the visual comparison between LKDA-Net and other models in the Synapse multi-organ segmentation task. UNETR exhibits discontinuities in segmentation boundaries for the pancreas, Swin-UNETR demonstrates erroneous segmentation in the pancreatic head region, and nnFormer suffers from incomplete segmentation of the kidneys. In contrast, LKDA-Net’s predictions align more closely with the ground truth, particularly in capturing subtle boundary structures, where its contour continuity and spatial consistency significantly outperform competing models. These observations validate the enhanced global context modeling capability of LKDA-Net’s large-kernel depthwise separable convolution attention (LKD Attention).

**Fig 6:**
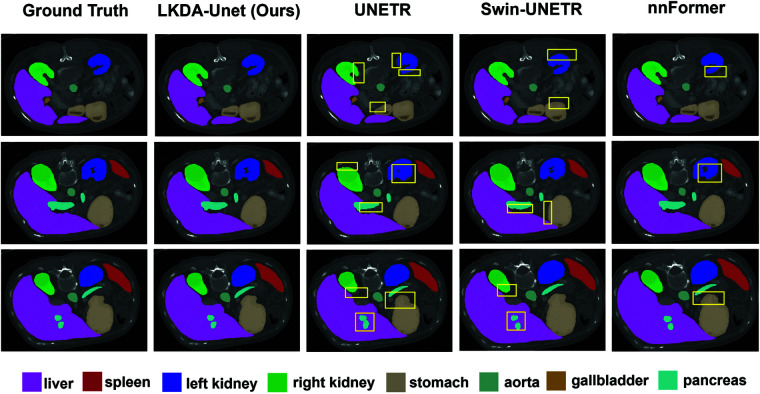
Comparison results of visualization on the BTCV dataset. Zoomed-in boxed regions highlight significant differences in segmentation quality. LKDA-Net exhibits superior contour alignment with the ground truth for small anatomical structures, such as the esophagus (ESO), gallbladder (GAL), and adrenal glands (RAG, LAG), while substantially reducing mis-segmentation artifacts.

Fig 6 shows the visual comparison between LKDA-Net and other models in the BTCV abdominal multi-organ segmentation task. Here, nnFormer and nnUNet display incomplete segmentation of the portal splenic vein (PSV) with boundary blurring, while Swin-UNETR fails to accurately reconstruct the morphology of the right adrenal gland (RAG). In contrast, LKDA-Net effectively integrates multi-scale features through its Skip Connection Fusion Module, preserving fine-grained anatomical details to enhance segmentation precision while significantly mitigating edge ambiguity.

### Ablation experiment

We conducted ablation studies on the LKDA-Net Block and the Skip Connection Fusion Module respectively. In the ablation studies, we evaluated the parameters and segmentation performance of different architectural configurations on the Synapse dataset. We adopted Swin-UNETR as the baseline model and conducted the following experimental configurations: (1) Replacing the Windowed Multi-Head Self-Attention (W-MSA) in Swin Transformer Blocks with our proposed LKD Attention (3 × 3 × 3 DWConv + 5 × 5 × 5 DWConv). (2) Using LKD Attention with larger kernels (5 × 5 × 5 DWConv + 7 × 7 × 7 DWConv). (3) Further scaling kernel sizes in LKD Attention (7 × 7 × 7 DWConv + 9 × 9 × 9 DWConv). (4) Substituting the MLP in Swin Transformer Blocks with our Inverted Bottleneck with Depthwise Convolutional Augmentation (DWCA). (5) Replacing Swin Transformer Blocks with LKDA-Net Blocks. (6) Combining LKDA-Net Blocks with the Skip Connection Fusion Module in the full architecture.

Table 6 shows the performance of adding the two modules proposed in this paper to the Swin-UNETR baseline model on the Synapse dataset. The experimental data indicate that the number of parameters of the Swin-UNETR model on the Synapse dataset is 62.83M, and the average DSC is 83.48%. When the Window Multi-Head Self-Attention (W-MSA) in the Swin Transformer Block of the Swin-UNETR model is replaced with the LKD Attention proposed in this paper, the number of parameters of the model is significantly reduced, and the average DSC is also improved. In addition, we conducted experiments with different configurations for the kernel sizes of the two Depthwise Convolutions (DWConv) in the LKD Attention . In the ablation study, we observed that the LKD Attention module achieved a mean Dice Similarity Coefficient (DSC) of 85.21% when employing a combination of 5 × 5 × 5 and 7 × 7 × 7 convolutional kernels, outperforming other configurations. This superiority can be attributed to two key factors: From the perspective of multiscale context capture, the 5 × 5 × 5 kernel effectively captures midrange contextual information within local regions, while the 7 × 7 × 7 kernel extends the receptive field to model global anatomical dependencies. Their cascaded design recursively aggregates multiscale features, enhancing the model’s capacity to represent complex organ boundaries and heterogeneous regions. From the standpoint of parameter efficiency and feature representation balance, larger 9 × 9 × 9 kernels further expand the receptive field, they introduce a significant parameter increase (50.13M) and risk incorporating redundant noise. Conversely, smaller 3 × 3 × 3 kernels fail to adequately cover global dependencies. The 5 × 5 × 5 + 7 × 7 × 7 configuration achieves an optimal balance between receptive field coverage and feature precision while maintaining a lower parameter count (43.44M).

**Table 6:** The ablation experiment data of the LKDA-Net model on the Synapse dataset. We exhibit the parameter number and model performance when our model is incorporated into the baseline, and present the performance of LKD Attention with different convolution kernel sizes.

Methods	Params (M)	Average DSC(%)
Swin-UNETR	62.83	83.48
Use LKD Attention (3 × 3 × 3 DWConv + 5 × 5 × 5 DWConv)	40.52	82.81
Use LKD Attention (5 × 5 × 5 DWConv + 7 × 7 × 7 DWConv)	43.44	85.21
Use LKD Attention (7 × 7 × 7 DWConv + 9 × 9 × 9 DWConv)	50.13	84.63
Use DWCA	64.23	84.81
Use LKDA-Net Block	44.73	86.23
Use LKDA-Net Block + Skip Connection Fusion Module	48.52	87.21

Furthermore, when the Multi-Layer Perceptron (MLP) in the Swin Transformer Block is replaced with the Inverted Bottleneck with Depthwise Convolutional Augmentation (DWCA) module in the LKDA-Net Block, both the number of parameters and the segmentation performance of the model slightly increase. This module not only improves performance metrics but also addresses the semantic gap by processing the encoder’s shallow local features and the decoder’s deep semantic features separately through grouped convolutions, combined with inverted bottleneck pointwise convolutions to adaptively calibrate feature weights. Furthermore, it employs 3 × 3 × 3 grouped convolutions and GELU activation functions to enhance feature extraction, preserving edge details and subtle structures. By leveraging grouped convolutions to reduce redundant computations, the module achieves efficient fusion with minimal parameter overhead.

Moreover, on the basis of adding the LKDA-Net Block to the Swin-UNETR, the Skip Connection Fusion Module is added to the decoder. Although the number of parameters of the model increases to 48.5M, the segmentation performance is improved to 87.21%. These results demonstrate that the LKDA-Net Block can not only reduce the number of parameters of the model by using LKD Attention but also extract multi-scale features with a larger receptive field to improve the segmentation performance. And the Skip Connection Fusion Module can effectively fuse the shallow local information at each stage in the encoder and the deep semantic information in the decoder, further improving the accuracy of segmentation.

## Discussion

To understand the limitations of LKDA-Net, we analyzed the segmentation results of it and other SOTA methods on the Synapse Dataset and BTCV dataset, with a focus on the cases where the Dice scores were the lowest among these methods. Table 7 presents the Dice scores of these failure cases. The failure cases of these methods exhibited significant consistency, as almost all methods showed relatively low segmentation accuracy in the same instances within the two datasets. Specifically, in the BTCV multi-organ segmentation task, U-Net and TransUNet demonstrated the lowest segmentation accuracy in the first case and had similar low performance in the second case. In contrast, Swin-UNETR and nnFormer had the lowest Dice scores in the second case while performing poorly in the first case. In this study, we compared a variety of model architectures, including the pure convolutional U-Net, the pure Transformer architecture nnFormer, and the hybrid CNN-Transformer frameworks TransUNet and Swin UNETR. Therefore, these limitations might not stem from the architectural design or training. We speculate that certain anatomical features or pathological characteristics may inherently pose difficulties for segmentation algorithms.

**Table 7:** Analysis of failure segmentation results from LKDA-Net and other methods on Synapse and BTCV datasets. These segmentation results are appraised based on the average Dice score for each case.

Methods	Synapse		BTCV	
	Case #1	Case #2	Case #1	Case #2
U-Net	66.32	71.22	61.27	65.12
TransUNet	63.31	67.72	58.27	61.11
Swin-UNETR	58.31	55.32	55.40	52.23
nnFormer	65.24	63.11	60.24	59.21
LKDA-Net	70.12	73.57	67.12	68.87

## Conclusions

We propose the LKDA-Net, which is an effective network architecture for 3D medical image segmentation. The design of LKDA-Net aims to model global relationships by simulating the self-attention mechanism of Transformers based on the LKDA-Net Block, so as to efficiently extract global features and then achieve accurate and efficient 3D volume segmentation. The encoder of LKDA-Net consists of the LKDA-Net Block and downsampling. Specifically, the Large Kernel Depthwise Convolution Attention (LKD Attention) in the LKDA-Net Block extracts multi-scale features by employing multiple large kernel depthwise convolutions. It recursively aggregates the contextual information within the receptive field and captures more effective features in deeper and larger receptive fields at the same time. The Inverted Bottleneck with Depthwise Convolution Augmentation (DWCA) in the LKDA-Net Block reduces the redundancy among channels while enhancing the feature expression ability by independently expanding and compressing the dimensions of each channel. The decoder utilizes the Skip Connection Fusion Module to further extract hierarchical visual feature representations and optimize the segmentation results. Inside it, effective operations such as group convolution are adopted to reasonably allocate features for fusion. The upsampling module improves the resolution of the feature map and retains key features through techniques like bilinear interpolation. We have conducted a comprehensive evaluation of LKDA-Net on multiple publicly available 3D medical image segmentation datasets. The results show that compared with the current Transformer models, LKDA-Net reduces the computational complexity while improving the segmentation performance.
